# Barriers and Facilitators of Screening for Sexually Transmitted Infections in Adolescent Girls and Young Women in Mombasa, Kenya: A Qualitative Study

**DOI:** 10.1371/journal.pone.0169388

**Published:** 2017-01-03

**Authors:** Ethel Avuvika, Linnet N. Masese, George Wanje, Juliet Wanyonyi, Benard Nyaribo, Grace Omoni, Anisa Baghazal, R. Scott McClelland

**Affiliations:** 1 University of Nairobi Institute of Tropical & Infectious Diseases (UNITID), Nairobi, Kenya; 2 Department of Epidemiology, University of Washington, Seattle, Washington, United States of America; 3 Student Services, Technical University of Mombasa, Mombasa, Kenya; 4 Mombasa County Department of Health, Mombasa, Kenya; 5 School of Nursing Sciences, University of Nairobi, Nairobi, Kenya; 6 Department of Medicine, University of Washington, Seattle, Washington, United States of America; 7 Department of Global Health, University of Washington, Seattle, Washington, United States of America; University of Toronto Dalla Lana School of Public Health, CANADA

## Abstract

**Objective:**

Young women bear the greatest burden of sexually transmitted infections (STIs), so it is important to identify and address barriers to STI screening in this population. We conducted a qualitative study to explore the feasibility of STI screening among adolescent girls and young women in Mombasa, Kenya.

**Methods:**

We conducted 17 in-depth interviews (IDIs) (8 with adolescent girls and 9 with young women) and 6 focus group discussions (FGDs) (4 with adolescent girls and 2 with young women, total 55 participants). The audio recordings for the IDIs and FGDs were translated and transcribed into English. Transcripts were independently reviewed by two researchers, and a set of codes was designed to help analyze the data using the content analysis approach. Data content was then analyzed manually and digitally using ATLAS.ti, and consensus was reached on central and specific emergent themes discussed by the research team.

**Results:**

Adolescent girls and young women in Mombasa, Kenya expressed willingness to participate in STI screening. A major incentive for screening was participants’ desire to know their STI status, especially following perceived high-risk sexual behavior. Lack of symptoms and fear of positive test results were identified as barriers to STI screening at the individual level, while parental notification and stigmatization from parents, family members and the community were identified as barriers at the community level. Uncomfortable or embarrassing methods of specimen collection were an additional barrier. Thus, urine-based screening was felt to be the most acceptable.

**Conclusion:**

Kenyan adolescent girls and young women seem willing to participate in screening for STIs using urine testing. Addressing stigmatization by parents, health care workers and the community could further facilitate STI screening in this population.

## Introduction

The transition from adolescence to adulthood is marked by several reproductive health challenges. In sub-Saharan Africa, these challenges are rarely addressed, frequently resulting in poor reproductive outcomes such as sexually transmitted infections (STIs) and unwanted pregnancies [1, 2]. Premarital sex and discussions about adolescent sexual health are often prohibited. Culture and religion play a major role in promoting adult discomfort in dealing with these issues [3–5]. Young people may be reluctant to seek information from parents due to concern that they will be judged to be engaging in prohibited activities [3]. However, due to urbanization and western influence, sexual and reproductive norms are rapidly changing, with youth becoming sexually active at a younger age [6–8]. There is urgent need to eradicate barriers that youth face in accessing reproductive health information and services [9].

Adolescent girls and young women represent a vulnerable population disproportionately affected by STIs. Globally, adolescent girls and young women 15–24 years old are the most affected [1, 10], with age under 25 years being the strongest predictor of STIs [11–14]. Other risk factors include high risk sexual behavior associated with alcohol and drug use [15], low levels of knowledge about STIs, perceived peer norms, non-use of condoms [16], and intergenerational relationships [17]. With the burden of disease and complications from STIs being higher among young women [18], it is important to identify and address barriers to STI screening, and develop interventions targeted to this population. In Kenya, only 7% of health facilities offer youth friendly services, suggesting that youth access to reproductive health is limited. In addition, the majority of health services, including STI screening, are not free [19].

Adolescents girls are an understudied population due to the ethical and legal challenges that limit their participation in research [20]. For example, in sub-Saharan Africa, few studies have assessed the barriers and facilitators of screening for STIs other than HIV for this age group. Screening for STIs is recommended because STIs can be asymptomatic [21]. Early diagnosis and treatment are useful in preventing the spread and sequelae of STIs. Improving adolescent girls’ participation in research is crucial in defining and developing interventions appropriate to this age group.

To explore adolescent girls’ and young women’s attitudes towards STI screening, we conducted a qualitative study to address the following questions: 1) What do adolescent girls and young women know about STIs? 2) Are adolescent girls and young women willing to be screened for STIs? 3) What are the facilitators and barriers to STI screening in this population? 4) Which STI screening method would they prefer?

## Methods

We conducted in-depth interviews (IDIs) and focus group discussions (FGDs) with adolescent girls 15–17 years old and young women 18–24 years old, enrolled in educational institutions in Mombasa County, Kenya. Mombasa County region is considered urban. The study was conducted between December 2012 and November 2013. Ethical approval was granted by the ethics committees at the Kenyatta National Hospital, Nairobi, Kenya (P228_04_2012) and the University of Washington, Seattle, Washington (42869C).

We sought permission to conduct the study from institutional heads at six institutions (five high schools and one university) with approval from the Director of Education, Mombasa County. Of these, four agreed to work with us, including one university and three high schools. These four institutions each had large student populations with representation from different socioeconomic strata, ethnic and religious backgrounds, allowing us to capture a wide range of views.

We used purposive sampling to allow us to obtain a socio-demographically diverse sample. We had ethical approval to conduct up to 20 IDIs and 8 FGDs as the target sample size for this study. This sample size was felt to be sufficient based on qualitative work by Halcomb and Sandelowski [22, 23]. Halcomb suggested dividing the groups by type of participant, in our case the age bracket. For each type of participant group, Halcomb recommended that a minimum of 2 FGDs should be conducted to achieve data saturation (no new information emerges from the discussion) [22]. We conducted 6 FGDs (4 with adolescent girls and 2 with young women, total 55 participants). Based on previous experience conducting individual interviews, we expected that 8–10 participants per participant type will be enough to reach data saturation (no new information emerges from the interview) [24]. We conducted 17 individual IDIs (8 with adolescent girls, and 9 with young women) as our sample.

We began by conducting sensitization activities at the institutions. These activities included presentations to adolescent girls and young women at their respective institutions through forums such as club days. At these initial meetings, we introduced the research team and provided background information to familiarize participants with statistics related to STIs in their local setting. We also presented the objectives and procedures of the study, and explained the informed consent process. The presentations were followed by question and answer sessions. Interested individuals were given informed consent forms and asked to take the forms home to discuss with their parents or guardians, family, and friends. Assent/consent forms were available in both English and Swahili. Willing participants were required to give their assent/consent for participation in an IDI or FGD by signing the informed consent form. Parental consent was required in addition to participant assent if the participant was a minor (below 18 years). The students interested in participating were required to return their completed form to the guidance and counselling teacher. The consenting process was completed within two weeks. Participants were then scheduled for either an IDI or FGD, depending on their preference. On the scheduled interview or FGD date, we collected participants’ socio-demographic information prior to initiating the interviews or FGDs. Participants also signed a confidentiality agreement before each FGD to ensure that the content of the discussion would not be discussed outside of the FGD. We had no way of verifying that the parents signed the consent forms. This concern was discussed with the local ethics committee, which felt that the strategy we employed provided a reasonable balance between risks and benefits.

Using semi-structured topic guides, we conducted 17 individual IDIs (8 with adolescent girls, and 9 with young women. The IDIs were conducted at the institutions or at the research clinic, depending on the subjects’ preference. Interviews were carried out in English or Swahili, and all participants were fluent in at least one of these languages. To promote candid responses and discussions, students were assured of anonymity and confidentiality. Preliminary analyses and ongoing adaptation of interview guide content were initiated during the interviews. Interesting themes that emerged in earlier interviews were explored further in subsequent interviews and discussions until saturation was achieved and no new information was emerging from the interviews and FGDs. Interviews lasted about 1 hour. Participants received KES 500 (USD 5) as transport reimbursement.

A total of 6 FGDs were conducted, (4 with adolescent girls and 2 with young women, total 55 participants). The procedures for FGDs were similar to those described for IDIs, including topic guides, languages, and ongoing adaptation of content. In addition, at the start of each FGD, participants were reminded that all content discussed in the FGDs was confidential. Focus group discussions lasted about 1.5 hours.

Interviews were conducted by G.W. and E.A. who were not known to the research participants prior to participating in the study. We audio-recorded all interviews and discussions and took manual field notes. The audio recordings for both the IDIs and FGDs were translated and transcribed into English. Data from the IDIs and FGDs were analyzed using content analysis approach, an approach widely used in qualitative research [25]. This involved multiple readings of the transcripts to capture context and meaning, followed by coding and categorization of recurring concepts and ideas. The transcripts were reviewed separately by two trained independent researchers (G.W. and E.A.) for text element and key word coding. A coding matrix of all categories of overarching themes was developed and codes compared and added or removed based on the agreement between the two investigators. This multiple coding ensures the qualitative analog of inter-rater reliability [25, 26]. After coding, the emerging themes were organized according to the objectives of the study. Recurring themes emerging from the FGDs were noted, and served to validate data obtained from the interviews. Data content was then analyzed manually and digitally using ATLAS.ti (version 5.0, Berlin, Germany), and consensus was reached on the central and specific emergent themes.

## Results

### Baseline characteristics

We enrolled 72 students in the study, 46 (63.9%) were adolescents and 26 (36.1%) were young women (Table 1). The median age of the adolescents was 17 years (interquartile range [IQR] 15–17) while that of the young women was 21 years IQR (20–23). Twenty eight (61%) of the adolescents and 18 (69%) of the young women reported being Christians, while the rest were Muslim. All the adolescents were single with no source of income. Twenty-five (96%) of the young women were also single, and 13 (50%) reported income from casual labor.

**Table 1:** Baseline characteristics of adolescent girls and young women.

Characteristics	Median (IQR) or Number (percent)	
	Adolescents (N = 46)	Young women (N = 26)
**Age** (years)	17 (16–18)	21 (20–24)
**Marital status**		
Single	46 (100)	25 (96)
Married	0 (0)	1 (4)
**Religion**		
Christian	28 (61)	18 (69)
Muslim	18 (39)	8 (31)
**Any children**	0	0
**Source of income**		
Unemployed	0 (0)	13 (50)
Casual laborer	0 (0)	13 (50)

Seven main themes regarding feasibility of STI screening among adolescents and young women were identified. (1) Knowledge about STIs, (2) Acceptability of STI screening, (3) Facilitators of screening for STIs, (4) Barriers to screening for STIs, (5) Suitable location to offer STI screening, (6) Preferred mode of STI screening and (7) Disclosure of STI screening results for adolescents girls

### Knowledge about STIs

Both adolescents and young women were able to accurately define STIs, and to specifically name STIs such as gonorrhea, chlamydia, herpes, chancroid, syphilis, and HIV. Most were able to describe the signs and symptoms of STIs such as vaginal itching, discharge, and odor, genital sores, lower abdominal pain, dysuria and dyspareunia. One participant noted that she was taught in primary school that syphilis can cause a rash and sores. For some participants, there was lack of awareness that STIs can be asymptomatic. One participant reported past treatment for STI, while most reported having a friend who had suffered from an STI.

I had a friend who had the disease. It was difficult for her because most of the time she was just complaining, when she moved around she felt something in her private parts. She was having difficulties. The discharge, she was experiencing some discharge which was very smelly so it became uncomfortable for her. When passing urine she was complaining, she had some sores around the private parts; sometimes she experienced lower abdominal pains. (21 year old, IDI)

Most participants correctly reported sexual intercourse as the main mode of STI transmission. Others noted that STIs can be passed from mother to child during pregnancy or during delivery. There were also misconceptions. Some participants thought that having sex during a menstrual period will result in an STI. In addition, some expressed the opinion that STIs can be transmitted via sharing of clothing, and kissing.

You know, some girls who are friends can share anything that they have. Now they can share even panties because they trust each other, they might transmit those STIs. (17 year old, IDI)

Is there any other way it can be transmitted? Through sharing of clothing, and bathing. (21 year old, IDI).

The participants felt that STIs are dangerous because they can lead to infertility, and some STIs like HIV can lead to death. One participant noted that viral STIs (such as herpes and HIV) are not treatable, so preventing them is better. Participants stated that prevention of STIs could be achieved through abstinence, use of condoms, maintaining high standards of hygiene, wearing underwear made of cotton, and not sharing clothes and towels. However, they noted that abstinence was rare because of peer pressure, access to pornographic material, and raging adolescent hormones. Some participants were adamant about not using condoms because they thought condoms have holes. In addition, they feared pregnancy more than STIs. As a result, they were more willing to use other methods of contraception to avoid pregnancy. Participants agreed that early detection of STIs could reduce or prevent damage to the reproductive system.

I think with today’s generation, it is hard to find people who abstain due to these programs in our televisions, these soaps. All these things (TV programs) influence people into early sexual intercourse which exposes them to STIs. (22 year old FGD)

I don’t trust those condoms you know at times you can use those condoms (Laughs) and maybe they have holes in them and you won’t know. So you can spread the diseases. (17 year old, FGD)

Most participants reported that they had received a large part of their basic reproductive health information and knowledge from their primary school teachers. Others reported having received most of their knowledge from high school and college lecturers, with emphasis being placed on HIV. Siblings, peers, media, and internet were mentioned as additional sources of information on STIs. A few mentioned that their mothers discussed STIs with them. However, parents, especially fathers, were a rare source of information because they were perceived to be more judgmental than siblings or peers. Their communication came largely as warnings to avoid pregnancy and HIV.

Sometimes in school, yes in school we are taught biology, we are taught STIs so that is how I learnt about them in biology yeah I had heard about them in my former primary school at class 7, we just learnt about ways of preventing those STIs but we didn’t go into much details. At the time the two that we learnt were gonorrhea and syphilis. (16 year old, IDI).

From the media. Yes, from school, also from friends. These days we have the internet; you can browse and get something about the diseases. We were taught, a long time ago. First in primary school, then in high school, then in the university. (20 year old, IDI)

However the majority of students acknowledged that the information provided by teachers and parents was insufficient, given the consequences associated with STIs.

It’s too little! (The information provided on STIs). They have to be open to their kids, talk to them freely. Then the community at large has to teach its people about the infections, how they can contract something, i.e. create awareness of the diseases. (21 year old, IDI)

### Acceptability of STI screening

Both the adolescent girls and young women reported willingness to be screened for STIs, including those who reported that they were not sexually active. When prompted, they confirmed that testing was a way of confirming that they did not have any infection. Others were willing to be tested because some STIs, such as herpes or HIV, are not solely transmitted through sex. They noted that even if someone was not sexually active, unless they got tested, they might not know if they are infected.

I will go (for the test) I will go. You know if you haven’t done something (sex) you are free to go. (17 year old, IDI)

I am willing to be tested, I know I haven’t done anything. So I will just get tested. (16 year old, IDI).

### Facilitators of screening for STIs

Knowing one’s status was a leading motivator for both adolescent girls and young women to seek STI screening services. The participants used the term ‘knowing one’s status’ to refer to the idea of knowing if one has any STI. This was not specific to a particular STI. The quote below captures the generally positive feelings of participants toward participating in STI screening.

You [might be] suffering from a disease but you don’t know which disease [it is]. So when you go for the test, you kind of get a clear picture. You find out what you are suffering from so you stop worrying about those other diseases that you [think] you are suffering from. (16 year old, FGD)

In the 18–24 year old women, screening for STIs following high-risk sexual behavior such as having sex without using a condom was felt to be important. However, this did not emerge as a theme in the younger age group.

I am willing (to be tested)…The first time I took the test was for HIV and I normally take it. Then the urine test just to see if I have gonorrhea, I normally take it. There was a time I had sex without protection that’s why I chose to do so (get tested). (21 year old, FGD)

Participants were willing to undergo STI testing, provided that the health center had adequate facilities, and privacy could be assured.

### Barriers to screening for STIs

A few participants reported that they would only participate in screening if they had symptoms of STIs, as illustrated by the following quote.

I don’t think I can go for testing unless I have the symptoms.That’s when I can go for testing. (20 year old, IDI)

There was widespread fear of seeking STI testing, as some of the adolescents felt that they would be judged to be engaging in sex by their parents.

I do not want my parents to know, because some parents will start saying what has this one thought of ‘till she went and got tested? Maybe she is having sex and now she is suspecting herself ‘till she went and got tested so that she can know how she is. (17 year old, IDI)

The young women reported stigmatization from health care providers when accessing STI screening services. Most were of the opinion that visiting a clinic, screening, and testing positive for STIs would lead care providers to conclude that the young woman was promiscuous and a prostitute. In addition, some feared the partner notification process. As a result, they sought treatment at local pharmacy shops. None of the participants were aware of any youth-friendly reproductive health centers where they could seek STI testing. They were only aware of youth centers where young people receive guidance and counselling, but stated that these centers lack STI testing facilities.

When it comes to young women testing, most young women fear, because they fear that they will be seen going to the clinic or that hospital to get tested for STIs. Actually the doctor or the person testing them will know that, Aaah! “This person has come here because they have been indulging in sex.” They will seem promiscuous, so that person may want to cover up and pretend that they have never been doing these things, because they don’t want people to know they are doing these things (sex). (21 year old, IDI)

Most of the adolescents felt that the community in general, and the religious community in particular, was not supportive of addressing adolescent sexual and reproductive health.

No. (Laughs) No, no. When you want to go to a pastor maybe like you have a religious problem but these thing like STIs, I can’t go to a pastor. (17 year old, FGD).

In my view, you can’t go and ask a sheikh about those things. If you talk about STIs the sheikh will start giving you chapters or verses from the Koran (laughs). So, there are so many verses he could read because you are addicted to these things. So I can’t. (16 year old, FGD)

Participants also mentioned distance to STI testing facilities as a barrier to testing. Although they preferred that such facilities be located away from the school, they were in agreement that if STI screening facilities were located too far away, many youth would not seek the services.

### Preferred mode of STI screening

Participants were aware of STI screening using different methods such as blood, genital swabs and urine samples. Blood sample collection was one of the most widely cited methods of STI screening. Vaginal examinations were described as the least preferred mode of screening due to discomfort and embarrassment. Both adolescent girls and young women preferred urine collection for STI screening, because it is non-invasive. However, one participant was concerned that the urine sample might not detect any damage to internal organs.

Urine, because, if you compare to HIV you have to be injected, i.e. they inject and get some blood sample. The other (pelvic exam) may take some discharge and that is not comfortable. Urine, there is no problem with the urine. (21 year old, FGD)

Aaaah I prefer that urine sample because I don’t want anyone to see how my body is. (17 year old, FGD)

### Suitable location to offer STI screening

While we recruited girls and young women at educational institutions, both groups reported that these were not the locations of choice for STI screening. Many participants noted that being seen going for STI screening was stigmatizing, and could lead to their identification as being sexually active and promiscuous.

I would prefer somewhere else, because in school people know you here and that’s when they start asking you why [are] you going to test, what have you done? But if you go somewhere else, people, they don’t know you. It’s not all people that will respect your option. So it’s better a place you are not known than in a place you are known. (17 year old, IDI)

I think because I am in this institution, and let’s say you have set up a tent there, and I will be going there. I come across my friends and they all know that’s the tent where we will all be tested for STIs. So everyone will know that you will be going there. They will be like, “That one has an STI.” It is all in our minds. It’s what we think, because it doesn’t mean that when you are going for an STI test it will come out that you are infected, because it’s all in our minds. But if you are going there and you meet your friend there, you change your route because you don't want suspicion. (21 year old FGD).

### Disclosure of STI screening results for adolescents girls

There were differing opinions among adolescents girls regarding disclosure of STI test results to parents. Some girls were willing to share results with their parents if the parents would pay for treatment. In contrast, others felt that disclosure of results would create a hostile relationship with their parents, who would now be aware that the girls were sexually active. These divergent opinions about disclosure to parents are illustrated by the following quotes.

Okay, if I have been tested and I have gonorrhea, the parents have to be informed. As for me, example, let’s say maybe I have gonorrhea. They have to be informed, because I cannot afford hospital bills and everything else. (16 year old, IDI)

Yes, it is better to not let your parents know, because some parents can decide, if they trusted you, they will say, “If you get the disease just forget about us”. If they know, they will really criticize you. You might even end up committing suicide. (17 year old, FGD)

## Discussion

This qualitative study of adolescent girls and young women in Mombasa, Kenya provides insight into this population’s knowledge and attitudes towards STIs and STI screening. Many adolescents and young women may be willing to participate in STI screening. A major facilitator for screening was participants’ desire to know their STI status. Among the women 18–24 years old, this was especially true following high-risk sexual behavior. Lack of symptoms and fear of positive test results were identified barriers to STI screening at the individual level, while parental notification and stigmatization from parents, family members and the community were identified as barriers at the community level. Uncomfortable or embarrassing methods of specimen collection presented an additional barrier, with urine sampling being the preferred mode of specimen collection.

The barriers and facilitators of STI screening reported in this study are similar to those reported by participants in Africa and other parts of the world [27–29], highlighting a potential set of cross-cutting concerns for adolescents and young women across a range of populations. Similar barriers across the studies include limited STI knowledge, especially the fact that STIs can be asymptomatic, pelvic exams as a mode of specimen collection, perceived judgmental service providers and affordability of health care services. Across these studies, participants were more likely to go for STI screening if the services were accessible, facilities were well equipped, confidentiality was guaranteed, and urine testing was offered.

The education curriculum in Kenya provides basic sexual and reproductive health education, including an overview of STIs. The majority of students were knowledgeable about STIs, transmission modes, methods of diagnosis, and means of preventing STIs. Some participants were able to associate signs and symptoms to a particular STI. However, some knowledge gaps were evident. For example, being asymptomatic was identified as a barrier to STI screening, as lack of symptoms was incorrectly considered a sign of being free of STIs [21]. And some believed that condoms may not be effective as they have holes. Improved sexual health education can be beneficial to adolescent girls and young women. For example, comprehensive sex education has been associated with delayed initial sexual activity and responsible sexual behavior in adolescents, reducing the risk of STIs [30]. In our sample of adolescent girls and young women in Mombasa, media, internet, and youth groups were mentioned as sources of information. These are powerful platforms that could be utilized to provide high quality sex education [31, 32].

Sex is a taboo subject in most African cultures, and discussion between parents and children about STIs is rare [4]. Although aunts and older siblings are often used as a proxy in providing communication on sex and reproductive health issues, greater emphasis should be placed on parental communication. Participants noted that parental communication on reproductive health matters came mostly in form of warnings against unplanned pregnancy. Meaningful discussions around sexual matters were limited suggesting possible cultural influences or gaps in parents’ reproductive health knowledge [33]. Good parental communication can influence a child’s sexual behavior and decisions, and should be encouraged [34, 35]. Parents ought to be empowered to discuss sex, birth control, pregnancy, and other sexual and reproductive health topics with adolescents.

An important strength of this study was our collection of views from a sizeable group of girls and young women in high schools and a university in Kenya. The IDIs provided insight into individual’s perspectives, while FGDs enabled us to focus on cultural norms. Moreover, most of the studies on barriers and facilitators to STI testing have been done in developed countries. In Africa, the focus has largely been on HIV testing. Hence these rich data add to the limited literature on reproductive and sexual health among adolescents and young women in sub-Saharan Africa.

This study also had limitations. First, we used non-probability (purposive) sampling of participants. Second, the religious and cultural influences in our study population may be different from other settings. Third, the institutions that participated in the study are located in an urban setting. These three factors may limit generalizability. In addition, limited access to free or cheap youth friendly reproductive health service centers in the country was an obstacle to access. Our experience with this study would seem to indicate that when such services were available, young women would be willing to screen for STIs. Finally, due to limited data in this area and the exploratory nature of our study, we did not initially utilize a conceptual framework in the development of our interview guides. However, based on findings from this study, an ecological model would be an appropriate choice for similar studies in the future. Nonetheless, we feel that the study has provided valuable information about the feasibility of providing STI screening to adolescents and young women in a resource-limited setting where this type of screening has not previously been the norm.

### Conclusion

Our study suggests that adolescent girls and young women are willing to be screened for STIs, with urine testing as the preferred method of screening. Improving sexual health education and addressing stigma from parents, heath care workers and the community could facilitate STI screening and other sexual health interventions in this population.
